# Simultaneous voxel‐wise analysis of brain and spinal cord morphometry and microstructure within the SPM framework

**DOI:** 10.1002/hbm.25218

**Published:** 2020-09-29

**Authors:** Michela Azzarito, Sreenath P. Kyathanahally, Yaël Balbastre, Maryam Seif, Claudia Blaiotta, Martina F. Callaghan, John Ashburner, Patrick Freund

**Affiliations:** ^1^ Spinal Cord Injury Center Balgrist University Hospital Zurich, University of Zurich Zurich Switzerland; ^2^ Wellcome Centre for Human Neuroimaging, UCL Queen Square Institute of Neurology University College London London UK; ^3^ Department of Neurophysics Max Planck Institute for Human Cognitive and Brain Sciences Leipzig Germany; ^4^ Department of Brain Repair and Rehabilitation, UCL Institute of Neurology University College London London UK

**Keywords:** brain and spinal cord template, multiparametric mapping, neuraxis, SPM, voxel‐wise analysis

## Abstract

To validate a simultaneous analysis tool for the brain and cervical cord embedded in the statistical parametric mapping (SPM) framework, we compared trauma‐induced macro‐ and microstructural changes in spinal cord injury (SCI) patients to controls. The findings were compared with results obtained from existing processing tools that assess the brain and spinal cord separately. A probabilistic brain‐spinal cord template (BSC) was generated using a generative semi‐supervised modelling approach. The template was incorporated into the pre‐processing pipeline of voxel‐based morphometry and voxel‐based quantification analyses in SPM. This approach was validated on T1‐weighted scans and multiparameter maps, by assessing trauma‐induced changes in SCI patients relative to controls and comparing the findings with the outcome from existing analytical tools. Consistency of the MRI measures was assessed using intraclass correlation coefficients (ICC). The SPM approach using the BSC template revealed trauma‐induced changes across the sensorimotor system in the cord and brain in SCI patients. These changes were confirmed with established approaches covering brain or cord, separately. The ICC in the brain was high within regions of interest, such as the sensorimotor cortices, corticospinal tracts and thalamus. The simultaneous voxel‐wise analysis of brain and cervical spinal cord was performed in a unique SPM‐based framework incorporating pre‐processing and statistical analysis in the same environment. Validation based on a SCI cohort demonstrated that the new processing approach based on the brain and cord is comparable to available processing tools, while offering the advantage of performing the analysis simultaneously across the neuraxis.

## Graphical abstract

This study presents a new approach for the simultaneous analysis of the brain and cervical spinal cord embedded in a unique SPM‐based framework incorporating pre‐processing and statistical analysis in the same environment. The proposed method yielded comparable results to more standard brain‐ or cord‐specific approaches when used to analyse quantitative MRI. It offers a single tool for any MRI studies investigating neurological disease with spinal cord involvement.

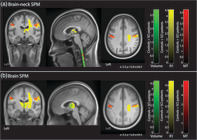

## 1 INTRODUCTION

A focal injury to the central nervous system produces widespread neurodegenerative changes, but also functional reorganisation across the neuraxis (Freund et al., 2019). Quantitative MRI revealed that after traumatic spinal cord injury (SCI), not only does the injured spinal cord undergo progressive neurodegeneration but with a time lag the cranial corticospinal tract and sensory‐motor cortices also show signs of enduring neurodegeneration (Freund et al., 2013).

Currently, analyses are performed separately in the brain and at the level of the spinal cord. The gold standard for assessing spinal cord atrophy is to measure changes in the cross‐sectional spinal cord area (SCA) at a single cervical level (C2/C3) (Papinutto et al., 2020) from T1‐ or T2‐weighted MRI acquisitions using the JIM software (Horsfield et al., 2010) or the spinal cord toolbox (SCT) (De Leener et al., 2017). In the brain, volume changes (based on the segmentation of T1‐weighted scans into tissue probabilities) or microstructural changes (via quantitative MRI, e.g., the multi‐parametric mapping [MPM] protocol, or diffusion weighted imaging) are analysed using software toolboxes such as statistical parametric mapping (SPM; https://fil.ion.ucl.ac.uk/spm) or FSL (https://fsl.fmrib.ox.ac.uk/). In particular, volume changes can be analysed using voxel‐based morphometry (VBM) (Ashburner & Friston, 2005) and microstructural changes can be analysed using voxel‐based quantification (VBQ) (Weiskopf et al., 2013). The MPM protocol provides indirect measures of myelin content, through the longitudinal relaxation rate (R1 = 1/T1) and magnetization transfer (MT), and of iron content, through effective relaxation rate (R2* = 1/T2*) (Leutritz et al., 2020; Tabelow et al., 2019; Weiskopf, Mohammadi, Lutti, & Callaghan, 2015).

However, there is no solution that enables the simultaneous assessment of changes occurring in the brain and cord. As a result, most imaging studies fail to implement and analyse the interactions between such remote areas across the central nervous system. Providing a tool that could assess the sequelae of a focal central nervous system (CNS) injury across the entire neuroaxis simultaneously would hold great potential to better understand the temporally and spatially distributed pathophysiological changes (Freund et al., 2016).

Anatomical templates are fundamental to group level comparisons because they naturally define a common space in which analyses can be performed. Templates typically either take the form of mean intensity images or of tissue probability maps (TPMs); the latter can further serve as anatomical priors in segmentation algorithms. A wide variety of brain templates have been developed, serving different purposes, but spinal cord templates are less common. Recently, a template that covers brainstem and spinal cord has been proposed (De Leener et al., 2018), using spinal cord specific tools that cannot be applied to the brain, so the conjoint analyses of the brain and cord is not possible. A principled way of constructing a template is to consider it as an unknown variable in a generative model of large MR datasets (Blaiotta, Freund, Cardoso, & Ashburner, 2018). In this context, a brain‐neck template has naturally been obtained by fitting the generative model to collections of images of the brain and spinal cord (Blaiotta et al., 2018).

The present study implements a brain‐neck template (covering brain and cervical spinal cord) and validates its use in the context of VBM (Ashburner & Friston, 2005) and VBQ (Weiskopf et al., 2013) analyses as implemented in the SPM framework. The extended SPM framework for brain and spinal cord analyses, named ‘SPM‐BSC’, offers the advantage of performing pre‐processing and statistics simultaneously in the brain and cervical spinal cord. In addition to voxel‐wise analyses in standard (group) space, cord‐specific metrics were also computed in native space. These include spinal cord area (SCA), anterior–posterior width (APW) and left–right width (LRW). Furthermore, mean values across the whole cervical spinal cord of the magnetization transfer saturation (MT), longitudinal relaxation rate (R1 = 1/T1) and effective transverse relaxation rate (R2* = 1/T2*) were also computed. The SPM‐BSC analysis was validated by assessing volume and microstructural changes in the brain and spinal cord in SCI‐patients relative to healthy controls and comparing the results with pipelines separately optimised for the brain (SPM‐BO for ‘brain only’), or from the SCT covering only the spinal cord.

## 2 MATERIALS AND METHODS

### 2.1 Participants and study design

MRI from 30 patients (age: 44.67 years ±16.72; 29 men) with chronic traumatic SCI (median months after injury 12.185, lower quartile 10.78, higher quartile 16.55) and 23 healthy controls (age: 36.87 years ±11.76; 13 men) were acquired at the University Hospital Balgrist between August 2011 and May 2015. There was no statistically significant age difference between the two groups (Mann–Whitney *U* test, z = −1.92, *p* = .055).

The exclusion criteria were: time since injury <2 months, pregnancy, head or brain lesions associated with spinal cord injury, pre‐existing neurological or medical disorders leading to functional impairments, mental disorder, or contraindications to MRI. All participants provided written informed consent prior to enrolment. The study protocols were in accordance with the Declaration of Helsinki and approved by the local ethics committee of Zurich the “Kantonale Ethikkommission Zurich” (EK‐2010‐0271). Patients underwent a comprehensive clinical assessment protocol, including the International Standards for Neurological Classification of Spinal Cord Injury (ISNCSCI) (Kirshblum et al., 2011) for motor, light touch, and pinprick score; the Spinal Cord Independence Measure (SCIM) (Catz et al., 2007); and additionally for tetraplegic patients the Graded Redefined Assessment of Strength, Sensibility (Table 1).

**TABLE 1:** Demographic information of patients with SCI included in the current study and corresponding clinical measurements

Participant	Age (years)	Time since injury (months)	Lesion completeness	AIS	Level of impairment (motor/sensory)	ISNCSCI LEMS	ISNCSCI UEMS	ISNCSCI pinprick	ISNCSCI light touch	SCIM
1	69	12.17	Incomplete	D	T11/T11	32	49	74	92	42
2	45	13.4	Incomplete	D	L3/L4	45	50	106	106	100
3	53	11.97	Incomplete	D	T10/T10	48	50	90	90	100
4	30	10.27	Complete	A	T10/T10	16	50	78	82	80
5	70	9.5	Complete	A	T7/T7	0	50	68	67	49
6	72	11.97	Incomplete	E	T3/T3	50	50	112	112	97
7	53	54.6	Complete	A	T3/T3	0	50	44	47	53
8	36	185.47	Complete	A	T12/T12	4	50	78	78	70
9	60	68.17	Complete	A	T1/T1	0	49	40	52	32
10	53	8.03	Complete	A	T9/T9	0	50	66	68	69
11	32	10.77	Incomplete	B	T11/T11	0	50	72	78	66
12	29	22.83	Incomplete	B	T6/T6	0	50	52	77	66
13	26	10.8	Complete	A	T4/T4	0	50	46	48	67
14	39	9.33	Complete	A	T7/T7	0	50	58	60	65
15	31	12.33	Incomplete	B	T4/T4	0	50	46	74	54
16	19	13.5	Complete	A	C6/C7	0	23	33	33	37
17	24	12.2	Incomplete	D	T1/C6	19	48	37	72	70
18	43	15.73	Complete	A	C6/C4	0	25	18	20	37
19	72	11.9	Incomplete	D	C6/C7	41	48	41	112	36
20	21	12.33	Complete	A	C6/C5	0	23	26	53	34
21	31	12.3	Incomplete	B	T1/C7	0	48	46	68	38
22	48	12.13	Incomplete	D	C5/C3	47	35	97	98	98
23	52	9.7	Incomplete	C	C7/C5	12	32	44	67	31
24	68	12.07	Incomplete	D	C3/C3	50	50	102	107	100
25	34	12.2	Complete	A	C7/C7	0	35	29	32	26
26	55	18.63	Incomplete	D	C3/C3	49	42	94	62	84
27	32	10.27	Complete	A	C6/C5	0	26	20	33	30
28	29	12.07	Complete	A	C5/C4	0	14	13	16	19
29	43	186.77	Incomplete	B	C6/C4	0	25	32	77	29
30	69	290.5	Incomplete	D	T1/C3	40	49	78	69	NA

### 2.2 Image acquisition

Participants underwent a 3D T1‐weighted MPRAGE (magnetization‐prepared rapid acquisition gradient echo) scan (whole‐brain including the cervical cord down to C5 level) on a 3 Tesla MRI scanner (Magnetom Verio [*n* = 35 subjects] or SkyraFit [*n* = 18 subjects] Siemens Healthcare, Erlangen, Germany) with the following parameters: field of view (FOV) of 224 × 256 × 176 mm^3^, isotropic resolution of 1 mm^3^, repetition time (TR) 2,420 ms, Echo time (TE) 4.18 ms, flip angle (α) 9°, inversion time 960 ms, and readout bandwidth of 150 Hz per pixel. The system was equipped with a 16‐channel radiofrequency (RF) receive head and neck coil and RF body transmit coil.

To assess microstructural changes, a multi‐parameter mapping (MPM) protocol, based on multi‐echo 3D FLASH sequences (Leutritz et al., 2020; Tabelow et al., 2019), was performed. These scans covered the whole brain and cervical spinal cord down to level C4 with 1 mm isotropic resolution and a field of view of 240 × 256 mm^2^ (matrix size of 240 × 256) with 176 partitions in a total scan time of 23 min. Parallel imaging with a speed up factor of 2 was used in the phase‐encoding direction (anterior–posterior) using a generalised auto‐calibration partially parallel acquisition algorithm (GRAPPA) and a partial Fourier acquisition with a 6/8 sampling factor was used in the partition direction (left–right) with a readout bandwidth of 480 Hz/pixel. Each set of echoes was acquired using a different TR and flip angle (α) to achieve images with either T1‐weighting: 25 ms/23°, PD‐weighting: 25 ms/4°, or MT‐weighting: 37 ms/9° with off‐resonance RF pulse (Gaussian shape with pulse length of 10 ms, flip angle of 500°, frequency 1.2 kHz and bandwidth of 192 Hz) prior to excitation. Echoes were acquired at six equidistant echo times (TE) from 2.46 ms to 17.22 ms for all weightings, with an additional echo at 19.68 ms for the PD‐weighted and T1‐weighted volumes.

### 2.3 Image analysis

VBM and VBQ analyses in SPM12 were extended to include cervical spinal cord (‘SPM‐BSC’) by incorporating a new probabilistic atlas of the brain and neck (Blaiotta et al., 2018). Validation of this approach was performed by analysing a cohort of 53 individuals comprised of 30 patients with chronic traumatic SCI and 23 healthy participants. The SPM‐BSC analysis was used to assess volume and microstructural differences within the brains and spinal cords of the SCI‐patients relative to the healthy controls. Results obtained with the proposed SPM‐BSC analysis were compared with those obtained using existing tools that separately assess either the brain or spinal cord only. Therefore, MRI data were analysed using:

- SPM‐BSC: the proposed VBM/VBQ pipeline implemented in SPM12 using a brain and spinal cord template enabling statistical analysis across the entire neuroaxis.
- SPM‐BO: the established VBM/VBQ pipelines available in SPM12 within the brain only.
- SCT: the spinal cord toolbox (SCT) (De Leener et al., 2017) covering only the spinal cord.

SPM analysis (SPM‐BSC and ‐BO) allows voxel‐wise statistical analysis to be conducted in MNI space, whereas SCT computes SC metrics in native space. In this study, in order to compare results from SPM‐BSC and SCT, SC metrics were additionally computed in native space using the SPM‐BSC method by applying the inverse deformation field.

### 2.4 Simultaneous analysis of the brain and spinal cord (SPM‐BSC)

The template incorporating brain and cervical spinal cord (named brain‐neck template) had been computed (Blaiotta et al., 2018) using a generative semi‐supervised modelling approach (Blaiotta, Jorge Cardoso, & Ashburner, 2016). The algorithm is based on a Gaussian mixture model of MR intensities, where the intensity distribution within each tissue type is assumed Gaussian, and the template encodes prior probabilities of belonging to each tissue (Blaiotta et al., 2016). A total of 12 weakly supervised components were optimised and, after algorithm convergence, they were combined into seven tissue maps based on visual inspection. In particular, a single component was used to model each of grey matter (GM), white matter (WM), cerebrospinal fluid (CSF) and fat; two components were used to model non‐neural tissues; three to model soft tissues and three to model a mixture of bone and air. Finally, the template was registered to the existing SPM12 tissue probability template using a 12‐parameter affine registration. This template covers the brain and spinal cord down to level C3.

#### 2.4.1 SPM‐BSC analysis in MNI space

**FIGURE 1:**
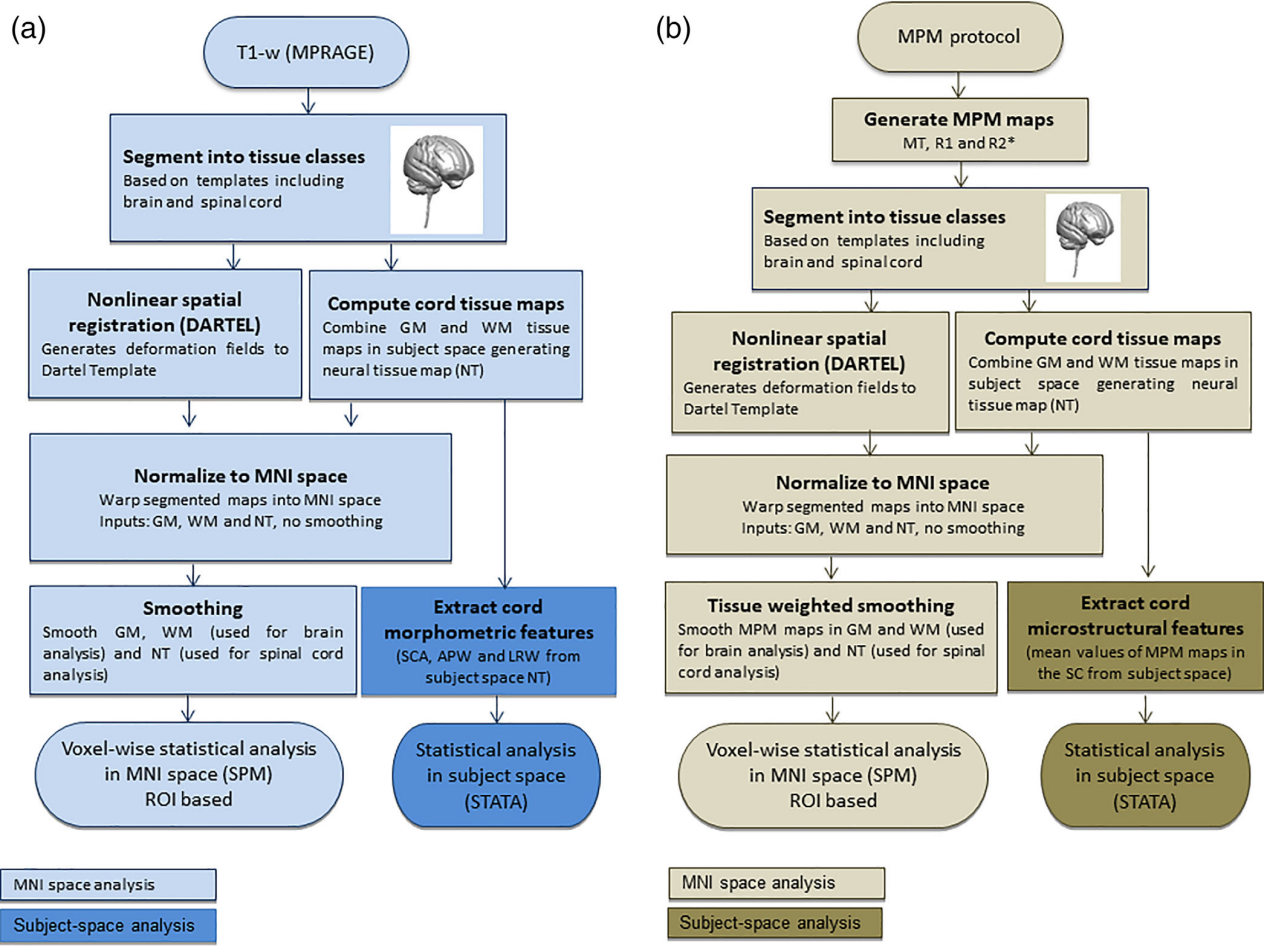
Image analysis: processing steps describing the new SPM‐BSC approach for VBM (a) and VBQ (b) analysis. MPRAGE, magnetization‐prepared rapid acquisition gradient echo); NT, neural tissue maps; WM, white matter; GM, grey matter

The brain‐neck template (Blaiotta et al., 2018) was used to segment the T1‐weighted structural MPRAGE images of each individual using the unified segmentation algorithm (Ashburner & Friston, 2005) in SPM12. The algorithm can use more than one Gaussian distribution to model intensities within each tissue class. Here, one Gaussian was specified for GM, WM, CSF and fat; two Gaussians were specified for non‐neural tissues and three Gaussians were specified for each of the other two tissue classes (soft tissues and a mixture of bone and air). To assess morphological changes in the spinal cord, the native‐space GM and WM tissue maps were combined to form a neural tissue (NT) class. Then, the GM, WM and NT maps were spatially normalised to MNI space with Dartel (Ashburner, 2007), and modulated by the Jacobian determinants of the deformations (Good et al., 2001). Finally, an isotropic Gaussian kernel of 6 mm full width at half maximum (FWHM) was applied to the modulated tissue maps (Figure 1a). The total intracranial volume (TIV) was computed from the sum of the GM, WM, and CSF volumes (Ridgway, Barnes, Pepple, & Fox, 2011).

The VBQ analysis (Draganski et al., 2011; Tabelow et al., 2019) was based on the quantitative metrics of MT, R1 and R2* derived from the MPM using the in vivo histology MRI (hMRI) toolbox (Tabelow et al., 2019). Inhomogeneity of the RF transmit field was corrected using UNICORT (Weiskopf et al., 2011). The VBQ analysis included segmentation of the MT map into GM, WM and CSF, covering brain and spinal cord, using the unified segmentation approach (Ashburner & Friston, 2005) and the brain‐neck template (as used when segmenting the MPRAGE for the VBM analysis). Segmenting the MT map ensures that the MPM maps and the tissue probabilities are aligned in native space. To assess microstructural changes in the spinal cord, NT maps were computed by again combining the GM and WM tissue maps from MT segmentation in native space. All tissue maps were transformed to MNI space using the Dartel algorithm (Ashburner, 2007). MPMs were warped to the MNI space using participant‐specific warping fields generated from the Dartel algorithm and finally smoothed using an isotropic Gaussian kernel filter with 6 mm FWHM using the VBQ smoothing approach with the corresponding tissue probability map (Draganski et al., 2011) (Figure 1b).

Smoothing the spinal cord MRI leads to undesired partial volume effects with CSF. Therefore, we investigated different smoothing kernels to identify the best scale space (Worsley, Marrett, Neelin, & Evans, 1996), for spinal cord analysis. We applied isotropic smoothing kernels with FWHM between zero (i.e., no smoothing) and 6 mm, with step of 1 mm, prior to the statistical analysis.

#### 2.4.2 SPM‐BSC analysis in subject space

To compute morphometric metrics in the spinal cord, such as SCA, APW and LRW, the native‐space NT tissue probability maps were thresholded at 0.5 to generate a binary mask of spinal cord. Next, an ellipse was fitted to the boundary of the mask in each transverse slice and the corresponding area (SCA) and main cord axes (LRW and APW) were extracted. Microstructural metrics were computed as the mean of the MT, R1 and R2* values within the masked region of the NT probability maps (computed from the MT map segmentation) covering cervical segments C1 to C3.

### 2.5 Brain analysis (SPM‐BO) in MNI space

MPRAGE anatomical images were analysed using the standard VBM approach, which segments and aligns the brain but does not account for the spinal cord. This procedure involved segmentation into GM, WM and CSF using unified segmentation (Ashburner & Friston, 2005) with the tissue priors released with the SPM12 software. The GM and WM tissue probability maps were subsequently warped into standard MNI space using Dartel, before being smoothed with an isotropic Gaussian kernel with 6 mm FWHM.

Multi‐parametric maps (MPMs) of MT, R1, and R2* were analysed using the standard voxel‐based quantification (VBQ) pipeline as implemented in the hMRI toolbox in SPM12. Briefly, MT maps were segmented using the tissue priors released with SPM12, which enabled the brains of the MPMs to be normalised to MNI space using Dartel, but could not align the cords of the group. Finally, the spatially normalised MPMs were smoothed using the VBQ approach with an isotropic Gaussian kernel filter with 6 mm FWHM (Draganski et al., 2011).

### 2.6 Spinal cord analysis in subject and template space using the spinal cord toolbox (SCT)

All MRI data were also analysed using the SCT software (De Leener et al., 2017). Here, the cervical C1‐C3 segments from T1‐weighted images and MT maps (from the hMRI toolbox) were segmented automatically using deep segmentation (McCoy et al., 2019). Segmented data were carefully inspected and manually corrected for misclassification if necessary (using FSL). Then, the generated spinal cord masks of each participant were registered to the MNI‐Poly‐AMU template (De Leener et al., 2018) using a combination of affine and nonlinear registrations, and the reverse deformation field (template to native) was applied to the WM and GM atlases, projecting them into the native space.

Next, morphometric parameters were extracted from the spinal cord in the segmented T1‐weighted image, across levels from C1 to C3 providing automatically APW, LRW and SCA. To perform microstructural analysis based on the MPM protocol, a pipeline was optimised based on SCT routines. Specifically the segmentation of MT maps provided a mask of the spinal cord which was aligned with all MPM maps in native space and used to extract microstructural parameters from the MT, R1 and R2* maps across levels C1 to C3. In addition, SCT smoothing along the centerline was applied to all MPMs using a Gaussian kernel with a standard deviation (σ) of 3 mm (De Leener et al., 2017), which corresponds with 5.88 mm FWHM. The SCT smoothing algorithm works by straightening the cord, applying a 1D Gaussian smoothing kernel and then un‐straightening the cord back into the original space (De Leener et al., 2017).

In order to provide voxel‐wise statistic in PAM50 space using SCT, the smoothed MPM maps were warped into this template space using the warping fields derived from the segmentation of the MT maps.

### 2.7 Statistical analysis

#### 2.7.1 Structural assessments of brain and spinal cord in MNI space

To assess morphometric and microstructural differences in the brain or spinal cord of SCI patients compared to healthy controls, a *t*‐test within the framework of the general linear model (GLM) in SPM was applied. Age, gender, scanner and total intracranial volume (TIV) were included as covariates of no interest to control for confounding linear effects in all GLMs (Barnes et al., 2010). A family‐wise error (FWE) correction using Gaussian random field theory was applied to account for multiple comparisons (Friston, Worsley, Frackowiak, Mazziotta, & Evans, 1994) within regions of interest (ROIs) using a threshold of *p* = .05 at the peak‐level. Only statistically significant results (*p* < .05) corrected for FWE are reported.

#### 2.7.2 Structural assessments of spinal cord in subject space

Additionally, spinal cord parameters in native space, such as SCA, APW, LRW and mean values from MT, R1 and R2*, extracted from both SCT and SPM‐BSC methods were used to assess differences between SCI‐patients and healthy controls using an ANCOVA in Stata (Stata Corp 13.0, College Station, TX). All statistical tests were corrected for age, gender and scanner by including them as cofactors of no interest. Only results where *p* < .05 are reported.

#### 2.7.3 Regions of interest

An ROI approach was applied based on the literature findings (Freund et al., 2013; Grabher et al., 2015). This was necessary to be equally sensitive to brain and cord changes using data from the different methods. The following ROIs were used in the voxel‐wise analysis of the brain: bilateral motor cortex M1 (precentral gyrus), bilateral somatosensory S1 cortices (postcentral gyrus), thalamus and corticospinal tract, which were extracted using the SPM Anatomy toolbox (Eickhoff et al., 2007). The ROI approach was also applied for the spinal cord analysis. For the analysis of SPM‐BSC data, a group‐specific spinal cord mask was computed by thresholding the average of the NT maps of all participants to include voxels with probability greater than 0.5 and further restricting this mask to the spinal cord from levels C1 to C3 (visually defined). For the SCT analysis, the ROI was defined from the PAM50_cord map limited to levels C1 to C3, as defined in the levels map provided with the SCT.

#### 2.7.4 Brain map reliability: Comparing SPM‐BSC versus SPM‐BO

To evaluate the reliability of the spatially normalised maps computed with the SPM‐BO and SPM‐BSC approaches, the intra‐class correlation coefficient (ICC) (McGraw & Wong, 1996) was calculated to define the degree of absolute agreement between the corresponding outputs from the two approaches. In particular, first, the mean values in each ROI (M1, S1, thalamus and corticospinal tract) were computed from each subject and map, pre‐processed using SPM‐BO and SPM‐BSC methods (i.e., mean value of MT map from each subject in the motor cortex [M1]), then the values from the two approaches (i.e., mean MT from SPM‐BO vs. SPM‐BSC) were compared using a two‐way mixed‐model, absolute agreement, and multiple raters ICC (ICC(A,1)) (McGraw & Wong, 1996).

According to the ICC guidelines (Koo & Li, 2016), ICC values can be partitioned to indicate poor (less than 0.5), moderate (between 0.5 and 0.75), good (between 0.75 and 0.9), and excellent (greater than 0.90) reliability between methods.

#### 2.7.5 Associations between spinal cord MPM maps and clinical outcomes

Spinal cord MRI data in the template spaces (from both SPM‐BSC and SCT analyses separately) were used to assess the associations between MPM measures and clinical outcomes (LEMS, light‐touch, pinprick and SCIM scores), using regression models in SPM12. All tests were corrected for age, gender, scanner and total intracranial volume (TIV) as covariates of no interest and family‐wise error (FWE) correction was applied to account for multiple comparisons (Friston et al., 1994) using a threshold of *p* = 0.05 at the peak‐level.

## 3 RESULTS

### 3.1 Structural changes in the brain using SPM‐BSC

**FIGURE 2:**
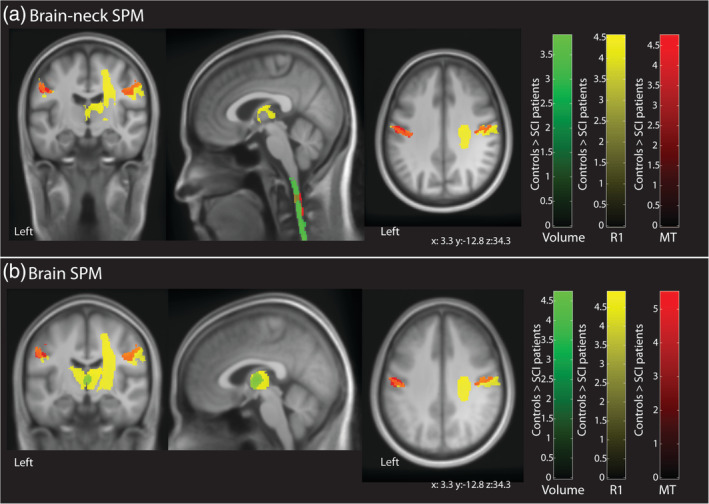
Overlay of statistical parametric maps (uncorrected *p* < .001, for illustrative purposes) showing morphometric and microstructural changes in SCI‐patients compared to healthy controls using data pre‐processed via the simultaneous brain and spinal cord (SPM‐BSC) approach in (a) and the standard brain approach in (b). Both pre‐processing methods showed myelin‐sensitive R1 reduction in the thalamus (yellow); myelin‐sensitive R1 and MT reduction in the bilateral sensory‐motor cortex (R1, yellow; MT, red); and myelin‐sensitive R1 reduction in right corticospinal tract in SCI‐patients compared to healthy controls (R1, yellow; MT, red). In addition, the SPM‐BSC approach (a) showed atrophy (green) and myelin‐sensitive MT reduction in the cervical spinal cord of SCI‐patients compared to healthy controls. The colour bar indicates the t score

The ROI‐based analysis revealed myelin‐sensitive R1 reduction (z‐score = 4.1, *x* = 9, *y* = −20, *z* = 18, *p* = .002) in the thalamus of SCI‐patients compared to healthy controls. Myelin‐sensitive R1 and MT reductions were observed in the primary motor (MT: *z*‐score = 4.0, *x* = 45, *y* = −17, *z* = 45, *p* = .018; R1: z‐score = 4.1, *x* = 45, *y* = −15, *z* = 45, *p* = .005) and sensory cortex (MT: z‐score = 4.3, *x* = 51, *y* = −17, *z* = 41, *p* = .011; R1: z‐score = 4.0, *x* = −48, *y* = −35, *z* = 47, *p* = .015) of SCI‐patients compared to healthy controls. Furthermore myelin‐sensitive R1 reduction (*z*‐score = 4.0, *x* = 24, *y* = −9, *z* = 41, *p* = .008) was observed in the right corticospinal tract of SCI‐patients compared to healthy controls (Figure 2a). All results are reported in Table 2.

**TABLE 2:** Results from region of interest (ROI) analysis using voxel‐based morphometry (VBM) and voxel based quantification (VBQ) from ‘Brain‐neck SPM’ and ‘Brain only’ methods

	ROI	z‐score	Peak *p*‐value (FWE corrected)	*x*	*y*	*z*
SPM‐BSC						
R1	Thalamus	4.1	.002	9	−20	18
MT	Motor cortex	4.0	.018	45	−17	45
R1	Motor cortex	4.1	.005	45	−15	45
MT	Sensory cortex	4.3	.011	51	−17	41
R1	Sensory cortex	4.0	.015	−48	−35	47
R1	Corticospinal tract	4.0	.008	24	−9	41
SPM‐BO						
VBM	Thalamus	4.2	.011	2	−11	5
R1	Thalamus	4.4	<.001	5	−15	15
MT	Motor cortex	4.8	<.001	45	−15	45
R1	Motor cortex	4.6	<.001	45	−15	45
MT	Sensory cortex	5.0	<.001	48	−15	44
R1	Sensory cortex	4.2	.007	−50	−35	50
R1	Corticospinal tract	4.1	.005	26	−8	8

### 3.2 Structural changes in the brain using SPM‐BO

The ROI‐based analysis revealed statistically significant atrophy (*z*‐score = 4.2, *x* = 2, *y* = −11, *z* = 5, *p* = .011) and myelin‐sensitive R1 reduction (*z*‐score = 4.4, *x* = 5, *y* = −15, *z* = 15, *p* < .001) in the thalamus of SCI‐patients compared to healthy controls. Myelin‐sensitive R1 and MT reductions were observed in the primary motor (MT: *z*‐score = 4.8, *x* = 45, *y* = −15, *z* = 45, *p* < .001; R1: *z*‐score = 4.6, *x* = 45, *y* = −15, *z* = 45, *p* < .001) and sensory cortex (MT: *z*‐score = 5.0, *x* = 48, *y* = −15, *z* = 44, *p* < .001; R1: *z*‐score = 4.2, *x* = −50, *y* = −35, *z* = 50, *p* = .007) of SCI‐patients compared to healthy controls. Furthermore, myelin‐sensitive R1 reduction (*z*‐score = 4.1, *x* = 26, *y* = −8, *z* = 8, *p* = .005) was observed in the right corticospinal tract of SCI‐patients compared to healthy controls (Figure 2b). All these results are reported in Table 2.

### 3.3 Brain map reliability: Comparing SPM‐BSC versus SPM‐BO

The ICC results are presented in Table 3. The ICC was moderate to excellent in all cases. Excellent ICCs (ICC ≥0.9) were observed in the motor cortex and sensory cortex of the R1 map; in the thalamus and corticospinal tract of the R2* map; and in the corticospinal tract of the R1 map. Good ICCs (0.75 ≤ ICC <0.9) were observed in M1 and S1 in the MT and volume maps; and in S1 of the R2* map. Moderate ICCs (0.5 ≤ ICC <0.75) were observed in the thalamus and corticospinal tract of MT and volume maps; in the thalamus of the R1 map; and in M1 of the R2* map.

**TABLE 3:** Intraclass correlation coefficient (ICC) using the mean values in the ROIs from data smoothed with 6 mm FWHM

ROI	MT	R1	R2*	Volume
M1	0.80	0.93	0.62	0.80
S1	0.88	0.98	0.85	0.88
Thalamus	0.56	0.56	0.95	0.56
Corticospinal tract	0.50	0.90	0.97	0.50

### 3.4 Spinal cord changes using voxel‐wise analysis in template space

**FIGURE 3:**
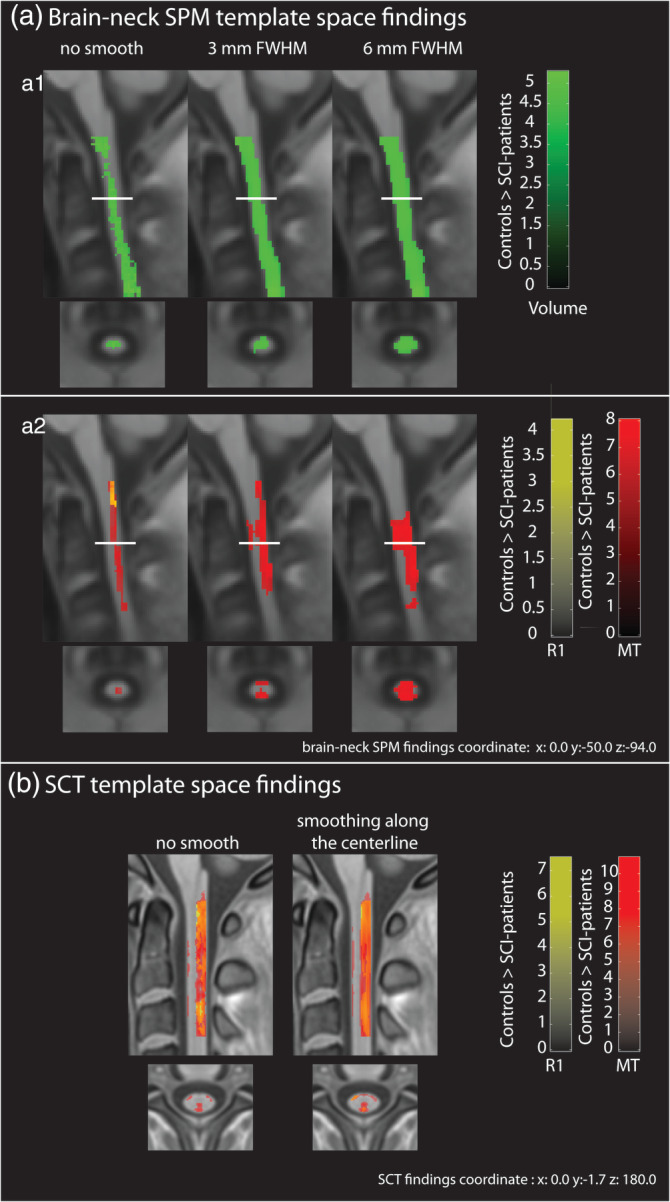
Overlay of statistical parametric maps (uncorrected *p* < .001, for illustrative purposes) showing statistically significant differences in SCI‐patients compared to healthy controls in the cervical spinal cord using different smoothing sizes and data analysed with SPM‐BSC (in a) and SCT (in b) approaches. The colour bar indicates the t score

Data pre‐processed using the SPM‐BSC approach showed spinal cord atrophy (from the VBM analysis) and decreased MT (form the VBQ analysis) in SCI‐patients compared to controls for all smoothing sizes (Table 4). Decreased spinal cord R1 was found in SCI‐patients compared to healthy controls only if no smoothing was applied (*z*‐score = 3.82, *x* = 0, *y* = −49.5, *z* = −78, *p* = .042, in brain‐neck template space) (Figure 3‐a2).

**TABLE 4:** Results from VBM and MT analyses of differences between patients and healthy controls in the spinal cord using different smoothing sizes

Smoothing size FWHM	*p*‐value (FWE‐corrected)	*Z* score
VBM		
No smoothing	.0035	4.63
1 mm	.0019	4.70
2 mm	.0007	4.70
3 mm	.0006	4.59
4 mm	.0004	4.56
5 mm	.0003	4.54
6 mm	.0003	4.50
MT		
No smoothing	<.0001	6.28
1 mm	<.0001	5.91
2 mm	.0001	5.18
3 mm	.0003	4.87
4 mm	.0006	4.65
5 mm	.0009	4.45
6 mm	.0016	4.21
R1		
No smoothing	.042	3.82

Data pre‐processed using the SCT approach showed reduced MT and R1 in SCI‐patients compared to healthy controls (Figure 3b) using both, unsmoothed (MT: *z*‐score = 7.01, *x* = 0, *y* = −3, *z* = 201, *p* < .0001; R1: *z*‐score = 5.94, *x* = −0.5, *y* = −3, *z* = 215, *p* < .0001, in PAM50 space) and smoothed data (MT: *z*‐score = 7.61, *x* = −0.5, *y* = −3, *z* = 201, *p* < .0001; R1: *z*‐score = 6.02, *x* = 0, *y* = −3, *z* = 213, *p* < .0001, in PAM50 space).

### 3.5 Spinal cord changes using extracted metrics in subject space

**FIGURE 4:**
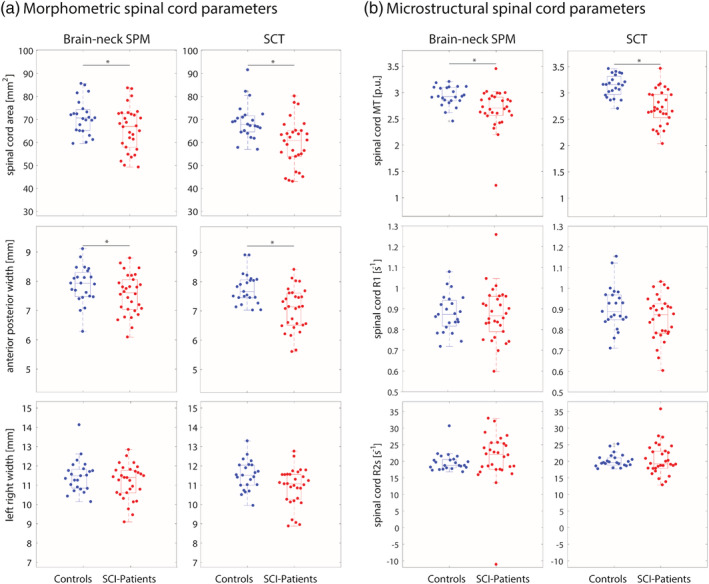
Structural changes in the cervical spinal cord applying SPM‐BSC and SCT tools: (a) morphometric analysis showing reduced cross‐sectional area and anterior posterior width in SCI‐patients compared to healthy controls (*p* < .05). (b) Microstructural analysis showing reduced MT in SCI‐patients compared to healthy controls (*p* < .05). The * indicates statistically significant differences (*p* < .05) between the connected groups

Morphometric analysis from data pre‐processed with the SPM‐BSC approach, revealed reduced SCA and APW in SCI‐patients compared to healthy controls (*p* < .05); while the LRW showed only a trend (*p* = .08) in the cervical spinal cord. Similarly, data pre‐processed with the SCT approach revealed reduced SCA and APW (*p* < .05) in SCI‐patients compared to healthy controls; while the LRW showed only a trend (*p* = .06) (Figure 4a and Table 5). Microstructural analysis from data pre‐processed with the SPM‐BSC approach, revealed reduced myelin‐sensitive MT and R1 (*p* < .05) in SCI‐patients compared to healthy controls; whereas measures of R2* in the cervical cord were not found to be different (*p* = .49). Similarly, data pre‐processed with the SCT approach revealed reduced myelin‐sensitive MT (*p* < .001) in SCI‐patients compared to healthy controls, but no differences were found in either the R2* maps (*p* = .55) or the R1 maps (*p* = .62) (Figure 4b and Table 5).

**TABLE 5:** Group comparison using spinal cord metrics computed from SPM‐BSC and SCT approaches

SC metrics	Healthy controls mean ± SD	SCI patients mean ± SD	Group differences (%)	*p*‐value
SPM‐BSC				
SCA (mm^2^)	71.05 ± 7.51	65.91 ± 9.61	7.23	.019
APW (mm)	7.87 ± 0.63	7.55 ± 0.68	4.06	.04
LRW (mm)	11.48 ± 0.85	11.17 ± 0.89	2.70	.08
MT (p.u.)	2.93 ± 0.19	2.69 ± 0.38	8.19	.004
R1 (s^−1^)	876.88 ± 90.38	873.51 ± 126.01	0.38	.047
R2* (s^−1^)	0.02 ± 0.003	0.02 ± 0.008	0	.49
SCT				
SCA (mm^2^)	69.10 ± 7.76	59.61 ± 10.08	13.73	.003
APW (mm)	7.76 ± 0.52	7.06 ± 0.72	9.02	.002
LRW (mm)	11.51 ± 0.77	10.91 ± 0.98	5.21	.059
MT (p.u.)	3.13 ± 0.21	2.72 ± 0.34	13.09	<.001
R1 (s^−1^)	907.01 ± 105.12	856.98 ± 106.66	5.51	.62
R2* (s^−1^)	0.02 ± 0.002	0.02 ± 0.005	0	.55

Abbreviations: APW, anterior–posterior width; MT, magnetization transfer saturation; R1, longitudinal relaxation rate; R2*, effective transverse relaxation rate; SCA, spinal cord area,.

### 3.6 Associations between spinal cord MPM maps and clinical outcomes

**FIGURE 5:**
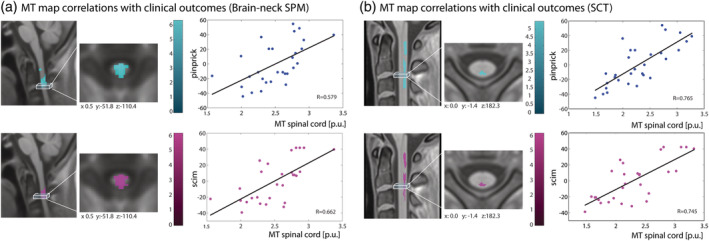
Associations between clinical measures and spinal cord MT maps pre‐processed using SPM‐BSC (in a) and SCT (in b); input data were smoothed with a 3 mm isotropic Gaussian kernel for SPM‐BSC and with sigma 3 mm for SCT approach using SCT tools

Association analysis using MPMs analysed with the SPM‐BSC approach showed that greater MT in the cervical cord was associated with better pinprick score (*z*‐score = 4.3, *x* = 0, *y* = −48, *z* = −108, *p* = .003, in brain‐neck template space) and better SCIM score (z‐score = 3.9, *x* = 2, *y* = −56, *z* = −104, *p* = .005, in brain‐neck template space) (Figure 5a). Similarly, MPMs analysed with the SCT approach showed that greater MT in the cervical cord was associated with better pinprick score (*z*‐score = 4.5, *x* = −1, *y* = −3, *z* = 202, *p* < .001, in PAM50 space) and better SCIM score (*z*‐score = 4.7, *x* = −1, *y* = −2, *z* = 207, *p* < .001, in PAM50 space) (Figure 5b).

## 4 DISCUSSION

In this study, a template covering the brain and the cervical spinal cord was validated within the SPM12 framework. This was done by comparing the results from the between‐group effect of SCI patients and healthy controls on qMRI data in the brain and in the spinal cord using the new SPM‐BSC analysis and established SPM brain and spinal cord toolbox analysis. In addition, good to excellent ICCs were observed in the majority of brain ROIs. Thus, the proposed SPM‐BSC approach enables analysis of equivalent data, sensitive to disease‐associated effects as brain or spinal cord, as established region‐specific analysis tools but with the advantage of being embedded within the simple unified SPM framework.

SCI patients exhibited lower R1 values in the thalamus, corticospinal tract and sensory‐motor cortex compared to healthy controls. Patients also displayed lower MT values than healthy controls in the sensory‐motor cortex. These changes are consistently found across analysis methods (see Table 2) and are in line with previous studies (Grabher et al., 2015; Seif et al., 2018), supporting the conclusion that SPM‐BSC approach is as sensitive as SPM‐BO for detecting group differences.

In addition, the ICC showed good to excellent values for most of the brain ROIs. Moderate ICCs were reported in the thalamus and corticospinal tract for MT and volume maps. These lower values might be due to discrepancies between the two templates, which were visually assessed by examining the difference between the standard and brain‐neck templates. The observed discrepancies can be attributed to the alignment strategy; in particular the standard brain template is smoother than the brain‐neck template since the latter used more precise alignment during the template generation. More direct comparison would be aided by using the same alignment strategy during the generation of the template and subsequent segmentation for both analysis approaches.

Voxel‐wise analysis in the spinal cord using the SPM‐BSC approach showed that SCI patients exhibited lower MT values than healthy controls, regardless of the size of the smoothing kernel used. However, R1 maps did not show the same results over all smoothing sizes and reduced R1 values were found only for unsmoothed data (Figure 3). This suggests that only focal differences were present in the R1 map within the cord and were obscured by partial volume effects resulting from the smoothing process. The SCT analysis using the PAM50 template showed reductions in MT and R1 values in SCI‐patients compared to healthy controls; and similar findings were found using smoothed data. The discrepancy between findings of reduced R1 between the two approaches in the smoothed case might be due to the smoothing algorithm applied: in the SPM‐BSC analysis, weighted isotropic Gaussian kernel smoothing was applied within the NT tissue class using the same VBQ approach applied in the brain (Draganski et al., 2011), enabling simultaneous assessment of the brain and neck using random field theory (Eklund, Nichols, & Knutsson, 2016). In contrast, in the SCT approach, a centerline smoothing algorithm was used, which works by straightening the cord, applying a 1D Gaussian smoothing kernel along its length and then un‐straightening the cord back into the original space (De Leener et al., 2017). The 1D nature of this latter approach may be more robust to partial volume effects, even within the cord, than the isotropic smoothing adopted in the VBQ approach. Nonetheless, both tools showed myelin sensitive MT changes that were consistent for all smoothing sizes. Further investigation of the impact of smoothing on voxel‐wise analyses in the spinal cord is needed.

Spinal cord native‐space analysis revealed atrophy and microstructural changes in SCI‐patients compared to healthy controls. In particular, both approaches (SPM‐BSC and SCT) showed atrophy in the spinal cord, more specifically in APW, and myelin‐sensitive MT reduction in SCI‐patients compared to healthy controls. However, different results were obtained for R1, where the SPM‐BSC approach showed differences between SCI‐patients compared to healthy controls, whereas in SCT this difference was not statistically significant. However, results from the analysis in PAM50 space (SCT) were more in line with those from the SPM‐BSC approach. Atrophy and microstructural changes in the spinal cord observed with the SPM‐BSC and SCT approaches in patients with SCI are in line those in the literature (Freund et al., 2019). This demonstrates that the SPM‐BSC approach can detect the same injury‐related differences between SCI‐patients and healthy controls as existing tools that are dedicated to either brain or cord. This is achieved without a loss of sensitivity and is the case for both native‐space spinal cord metrics and template‐space voxel‐wise analyses.

The association analysis between spinal cord microstructure and clinical disability showed that higher MT values in the spinal cord were associated with better sensory and motor outcome. The SPM‐BSC approach was able to replicate the associations between spinal cord microstructure and functional impairment in SCI‐patients that were identified by the SCT analysis, though the latter did show stronger correlation (higher R values). This might again be due to the different smoothing approaches used by the two methods, that is, SCT uses an adaptive Gaussian kernel oriented along the spinal cord centreline (De Leener et al., 2017). There are no standards for the correct shape and size of smoothing kernel to be used in the spinal cord, warranting further investigation on this topic.

Several limitations must be considered when using the new SPM‐BSC approach. First, the brain‐neck template covers only cervical spinal cord until level C3. However, a template spanning more spinal cord levels could be generated using the same algorithm (Blaiotta et al., 2018) by including MRI data covering more spinal cord segments. This will be the focus of future work. Furthermore, the GM/WM classification in the spinal cord is sub‐optimal. As a result, the probability maps of GM and WM were combined in the present study to generate a probability map of NT, which is a non‐specific measure of spinal cord volume. In future, better GM/WM classification within the spinal cord might be achieved by combining ultra‐high resolution spinal cord data and high‐resolution brain MRI during template creation. Lastly, results from data pre‐processed with the standard brain‐based SPM approach showed slightly higher z‐scores (see Table 3), which could be due to differences in alignment due to the templates and segmentation inputs used.

## 5 CONCLUSION

This study presents a new approach for the simultaneous analysis of the brain and cervical spinal cord in the SPM framework. The proposed method yielded comparable results to more standard brain‐ or cord‐specific approaches when used to analyse quantitative MRI. It offers a single tool for any MRI studies investigating neurological disease with spinal cord involvement. Validation based on comparing SCI‐patients and healthy controls showed that the SPM‐BSC analysis is as sensitive as available tools for detecting changes occurring in the brain and spinal cord separately. Template improvement, including better classification of GM/WM in the spinal cord and covering more spinal cord levels, will be the subject of future investigations. This approach provides a means of assessing brain and spinal cord interactions in health and disease.

## CONFLICT OF INTEREST

The authors report no conflict of interest.

## Data Availability

The data that support the findings of this study are available on request from the corresponding author. The data have not been made freely available on the internet due to privacy or ethical restrictions.
